# Study of Sural Nerve Complex in Human Cadavers

**DOI:** 10.5402/2013/827276

**Published:** 2013-12-16

**Authors:** S. R. Seema

**Affiliations:** Department of Anatomy, ESIC Medical College & PGIMSR, Rajajinagar, Bangalore, Karnataka 560094, India

## Abstract

*Aim*. The sural nerve complex (SNC) consists of four named components: medial sural cutaneous nerve (MSCN), lateral sural cutaneous nerve (LSCN), peroneal communicating nerve (PCN), and sural nerve (SN). The formation and distribution of the sural nerve vary in different individuals. SN is universally recognized by surgeons as a site for harvesting an autologous nerve graft. The nerve is widely used for electrophysiological studies. Hence the study of sural nerve complex was taken up. *Method*. SNC was observed by dissecting 100 lower limbs in the department of anatomy at three different medical colleges, over a period of 10 years. *Result*. Typical SN was observed in 60% of the cases. MSCN was present in all the cases; in 15% of the cases the MSCN followed an intramural course. LSCN was present in 80% of the cases. PCN was present in 70% of the cases and in most of the cases calibre was larger than that of MSCN. *Conclusion*. The knowledge about the variation in the origin and course of the SN is important in evaluating sensory axonal loss in distal axonal neuropathies and should be borne in mind by clinicians and surgeons.

## 1. Introduction

Sural nerve is one of the cutaneous nerves of the lower limb. It is a branch from tibial nerve (TN) in the popliteal fossa, descends between the two heads of the gastrocnemius muscle, and pierces the deep fascia in the middle third of the posterior surface of the leg. It is usually joined by the peroneal communicating nerve (sural communicating nerve) which is a branch of common peroneal nerve (CPN) [1, 2]. Many authors describe that sural nerve is formed by the union of medial sural cutaneous nerve with the peroneal communicating nerve [3–13]. Some other authors describe that sural nerve is formed by the union of medial sural cutaneous nerve with the lateral sural cutaneous nerve [14–17]. Because of these controversies the term sural nerve complex was coined by Ortiguela, which includes medial sural cutaneous nerve, lateral sural cutaneous nerve, peroneal communicating nerve, and sural nerve [6].

Medial sural cutaneous nerve originates from the tibial nerve in the popliteal fossa. It descends between the two heads of gastrocnemius muscle, deep to deep fascia covering the muscle. It becomes superficial by piercing the deep fascia at the junction of middle and distal thirds of the leg. The nerve lies usually medially sometimes laterally to the short saphenous vein. The nerve joins the peroneal communicating nerve to form sural nerve. When there is no communication between medial sural cutaneous nerve and peroneal communicating nerve, the medial sural cutaneous nerve supplies the lateral surface of the leg and gives off lateral branch to the heel and continues as lateral dorsal cutaneous nerve.

Lateral sural cutaneous nerve originates from common peroneal nerve in popliteal fossa; it descends between deep fascia and lateral head of gastrocnemius muscle; at the middle of the calf it pierce is deep fascia to become subcutaneous.

Peroneal communicating nerve originates in the popliteal fossa either from lateral sural cutaneous nerve or directly from the common peroneal nerve. The nerve communicates with the medial sural cutaneous nerve to form sural nerve.

The sural nerve is a sensory nerve of the lower limb that supplies the lower posterolateral part of the leg and lateral part of the dorsum of the foot. It is generally described as a sensory nerve but may contain motor fibres [18–20]. The sural nerve is universally recognized by surgeons as a site for harvesting an autologous nerve graft [15]. The nerve is widely used for electrophysiological studies. The formation and distribution vary in different individuals. The sural nerve is the most frequent donor nerve used for peripheral nerve grafting. Despite the widespread use of the sural nerve, there is scant attention reported in the literature about associated donor site problem. The peroneal communicating nerve is readily accessible to surgical harvest as it lies superficially. When there is a situation requiring limited length of nerve graft material, the peroneal communicating nerve alone can be harvested and medial sural cutaneous nerve can be preserved and associated symptomatic neuroma of the sural nerve will be diminished [6].

## 2. Material and Method 

Over a period of 10 years (from years 2001 to 2011) 100 embalmed adult lower limbs were dissected, which were available in the Department of Anatomy (Bangalore Medical College, Sri Devaraj Urs Medical College and M. S. Ramaiah Medical College). Of which 86 lower limbs were from male and 14 were from female cadavers. In all the limbs the components of sural nerve complex were identified and traced up to its origin and distally till the lateral malleolus. Lengths of the nerves were measured, and the data obtained were recorded in order, analyzed, and compared with that of previous workers.

## 3. Results

**Figure 1:**
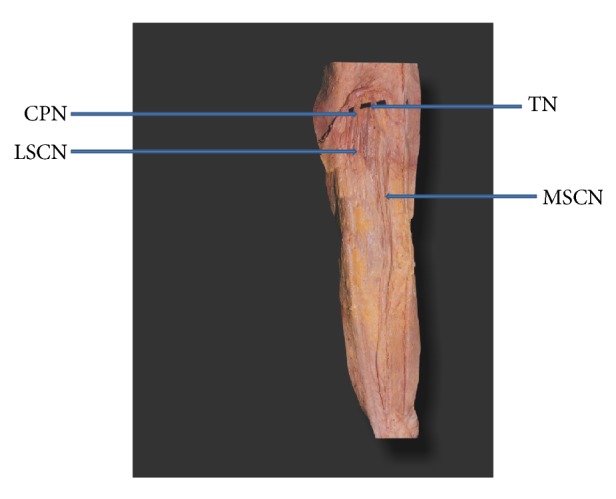
Medial sural cutaneous nerve continuing as sural nerve (MSCN: medial sural cutaneous nerve. LSCN: lateral sural cutaneous nerve. TN: tibial nerve. CPN: common peroneal nerve).

**Figure 2:**
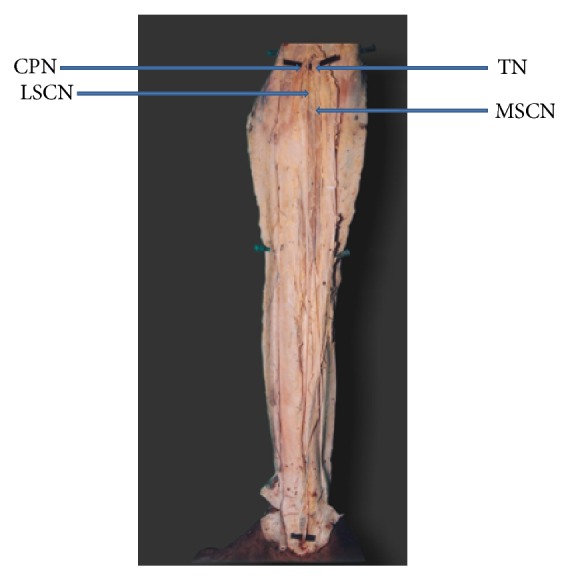
Lateral sural cutaneous nerve continuing as sural nerve (MSCN: medial sural cutaneous nerve. LSCN: lateral sural cutaneous nerve. TN: tibial nerve. CPN: common peroneal nerve).

**Figure 3:**
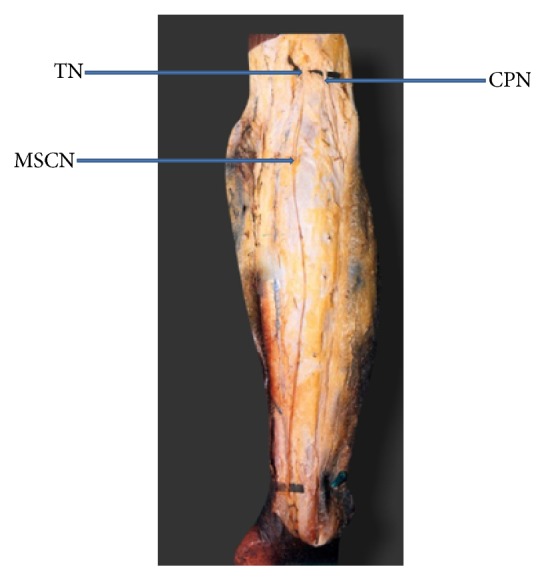
Both peroneal communicating nerve and lateral sural cutaneous nerve are absent (MSCN: medial sural cutaneous nerve. TN: tibial nerve. CPN: common peroneal nerve).

The typical sural nerve was formed by the union of peroneal communicating nerve (PCN) with medial sural cutaneous nerve (MSCN) in 60% of the cases. In 40% of the cases there was no communication between MSCN and PCN. In these cases PCN was considered as absent. In 39% of the cases MSCN alone continued as sural nerve (Figure 1). In 1% of the cases lateral sural cutaneous nerve (LSCN) continued as sural nerve and MSCN ended by giving calcaneal branches (Figure 2). Both PCN and LSCN were absent in 5% of the cases (Figure 3).

**Figure 4:**
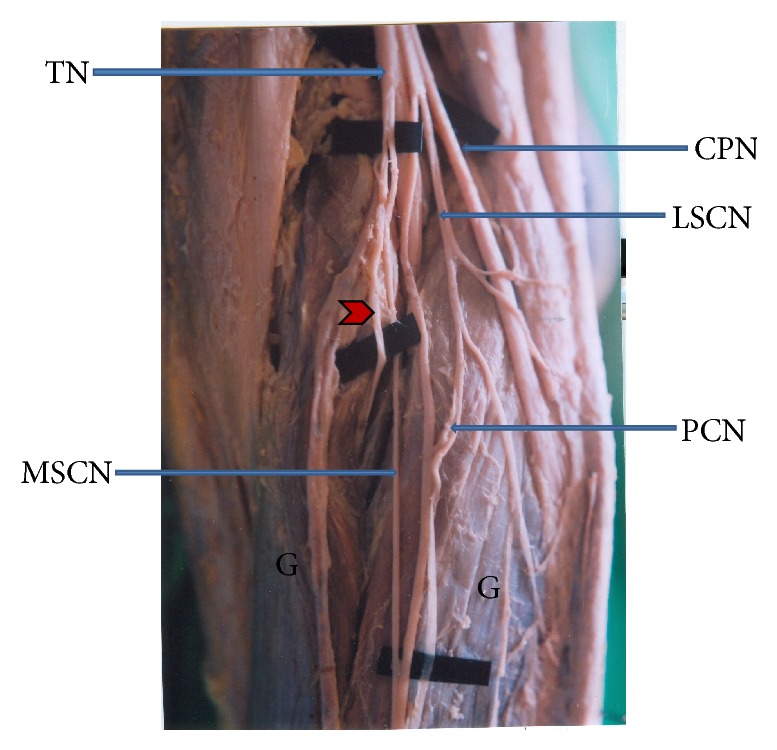
Medial sural cutaneous nerve originating from nerve to medial head of gastrocnemius muscle (MSCN: medial sural cutaneous nerve. LSCN: lateral sural cutaneous nerve. TN: tibial nerve. CPN: common peroneal nerve. PCN: peroneal communicating nerve. G: two heads of gastrocnemius muscle. Arrow head shows nerve to medial head of gastrocnemius muscle).

**Figure 5:**
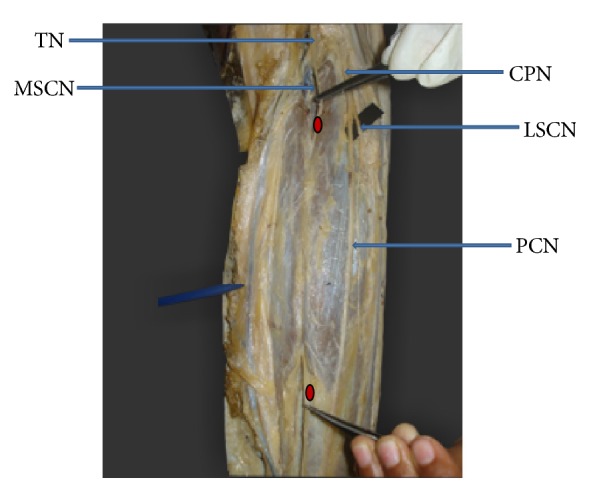
Intramuscular course of medial sural cutaneous nerve (MSCN: medial sural cutaneous nerve. LSCN: lateral sural cutaneous nerve. TN: tibial nerve. CPN: common peroneal nerve. PCN: peroneal communicating nerve. Bullet shows entry and exit of MSCN through gastrocnemius muscle).

In 4% of the cases MSCN was a branch from nerve to medial head of gastrocnemius muscle (Figure 4). In 11% of the cases MSCN showed an unusual intramuscular course. Here the nerve descends deep to two heads of gastrocnemius muscle instead of passing superficially to the muscle (Figure 5). In 1% of the cases MSCN pierced the lateral head of gastrocnemius muscle to become superficial.

In 40% of the cases PCN and LSCN arose by a common trunk. This trunk bifurcated into PCN and LSCN over the lateral head of the gastrocnemius muscle. PCN came as a terminal branch of LSCN in 16.6% of the cases. PCN arose directly from the common peroneal nerve, superior to the origin of the LSCN in 20% of the cases. In 5% of the cases PCN was the only branch of the common peroneal nerve to the posterior aspect of the leg, LSCN being entirely absent.

**Figure 6:**
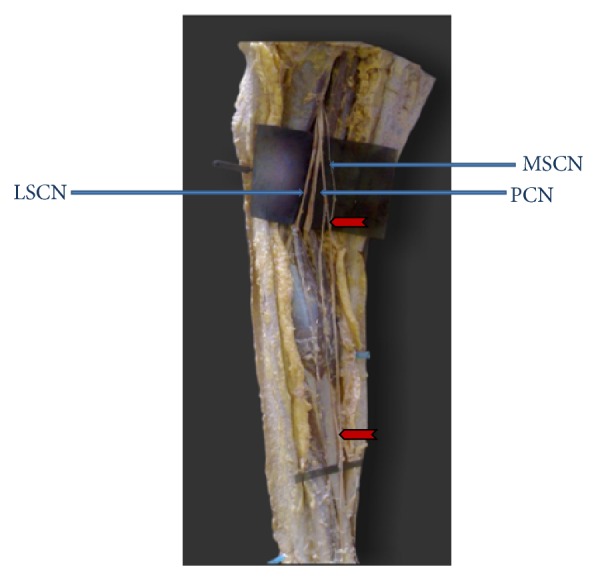
Peroneal communicating nerve communicated with medial sural cutaneous nerve at different levels (MSCN: medial sural cutaneous nerve. LSCN: lateral sural cutaneous nerve. PCN: peroneal communicating nerve. Arrow heads show different levels of communication).

**Figure 7:**
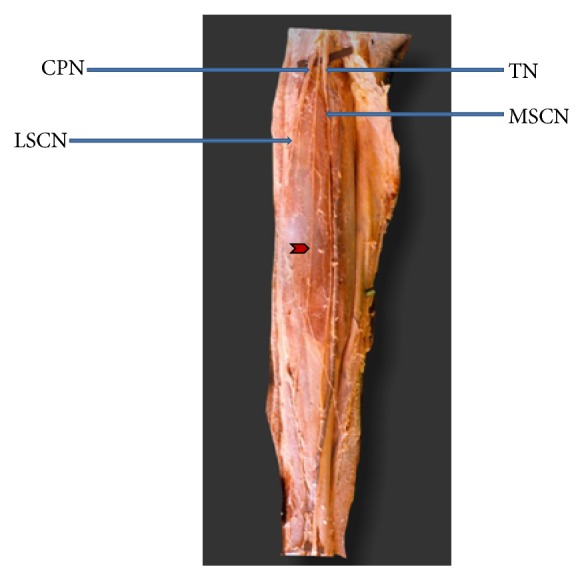
Arrow head shows additional branch from common peroneal nerve which did not communicate with medial sural cutaneous nerve (MSCN: medial sural cutaneous nerve. LSCN: lateral sural cutaneous nerve. TN: tibial nerve. CPN: common peroneal nerve).

It was observed that PCN divided into two branches and communicated with MSCN at different levels in 4% of the cases (Figure 6). In 1% of the cases apart from PCN, another branch from the LSCN also communicated with MSCN, distal to the communication between PCN and MSCN. In 2% of the cases the common peroneal nerve gave LSCN and another branch superior to the origin of LSCN. This additional branch did not communicate with medial sural cutaneous nerve (Figure 7).

Of the total number of typical sural nerves observed (60 cases), the union of PCN with MSCN was found in the distal 1/3 of the leg in 63.3% of the cases, middle 1/3 of the leg in 33.3%, and the popliteal fossa in 3.4%.

Length of the medial sural cutaneous nerve ranged from minimum 6 cms to maximum 43 cms, peroneal communicating nerve from minimum 2.5 cms to maximum 39 cms, lateral sural cutaneous nerve from minimum 8.5 cms to maximum 40 cms, and sural nerve from 3.5 cms minimum to 39.5 cms maximum.

## 4. Discussion

The typical sural nerve is formed by the union of medial sural cutaneous nerve with the peroneal communicating nerve. Few authors have reported that sural nerve is a branch of tibial nerve in the popliteal fossa and is usually joined by peroneal communicating nerve arising from common peroneal nerve [1, 21]; these authors considered the medial sural cutaneous nerve itself as sural nerve. When there is no communication between medial sural cutaneous nerve and peroneal communicating nerve, medial sural cutaneous nerve itself continues as sural nerve. Most of the studies reported this variation. The incidence of lateral sural cutaneous nerve continuing as sural nerve is rare [3, 5, 8, 11]. Summary of the incidence of formation of the typical sural nerve and medial and lateral sural cutaneous nerves continuing as sural nerve is presented in Table 1 and Table 2 summarizes the presence or absence of MSCN and LSCN.

The origin of the peroneal communicating nerve has led to a lot of controversies. Many authors describe it as a branch of lateral sural cutaneous nerve and few others describe the peroneal communicating nerve in majority of the cases arising alone or with the lateral sural cutaneous nerve from the common peroneal nerve [3].  Table 3 presents the origin of PCN.

“The name peroneal communicating can only be given to that nerve which unites with the medial sural cutaneous nerve to form the sural nerve.” At times one can see a nerve which has the course and position of typical peroneal communicating nerve, but it does not unite with medial sural cutaneous nerve. Rather, it supplies an area of the skin and subcutaneous tissue of the distal posterior part of the leg. This nerve is purely cutaneous and thus it must be considered as only a cutaneous terminal of the lateral sural cutaneous nerve [5].

Extensive work on sural nerve using electrodes found motor fibres in the sural nerve [18]. Cases of anomalous innervation of abductor digiti quinti muscle of the foot via sural nerve are recorded and showed compound muscle action on stimulation of sural nerve and medial sural cutaneous nerve, but not on stimulation of peroneal nerve [19]. Sekiya worked on communication between the sural nerve and tibial nerve and stated that there are three types of communications Y, U, and N. N type contains motor fibres which are derived from sural nerve. He also suggested that the medial sural cutaneous nerve fibres are essential for the skin and deep structures of the ankle and heel rather than the skin of the lateral side of the fifth toe and he designated medial sural cutaneous nerve fibres as T fibres [20]. Kim et al. studied the relative contribution of medial sural cutaneous nerve and peroneal communicating nerve to sural nerve by conduction study and showed that the main contributor to sural nerve was found to be the medial sural cutaneous nerve [9]. Many authors stated that medial sural cutaneous nerve takes a transmuscular course or is deeply embedded on the dorsal surface of gastrocnemius muscle. Since the nerve passes through the muscle, it is likely that it may give branch to gastrocnemius muscle. The abnormal course of the nerve may account for calf pain on contraction of gastrocnemius muscle or altered sensation over the area of distribution [3, 22–24]. In our study 11 cases of intramuscular course of medial sural cutaneous nerve were noted. Hwang et al. studied the morphometry of motor branches of tibial nerve. They found that tibial nerve innervating medial head of gastrocnemius muscle gave off medial sural cutaneous nerve in 30% of cases [25]. In our study it was 4%.

Entrapment of the sural nerve was reported due to posttraumatic scar tissue beneath the deep fascia of gastrocnemius, peroneal nerve sheath degeneration, calcaneocubiod joint capsule degeneration, and Achilles tendonitis [23, 24, 26]. Bergman et al. stated that sural nerve pierced medial head of gastrocnemius muscle before joining the peroneal communicating nerve [27]. In our study the medial sural cutaneous nerve pierced lateral head of gastrocnemius muscle before joining the peroneal communicating nerve. This can be one of the sites for entrapment.

Ortiguela et al. [6] and Coert and Dellon [15] found that the union of the medial sural cutaneous nerve with peroneal communicating nerve was in the distal 1/3 of the leg in the majority of the cases. Huelke found 75% of the cases in the distal half of the leg [5]. Eid and Hegazy found in 62% of the cases that site of union between these two nerves was in the lower one-third of the leg and ankle region [2]. William's study reported the majority in the superior 1/3 of the leg [3]. Uluutku et al. found that in 81.8% of the cases the union was at the middle 1/3 of the leg [7]. Mestdagh et al. found that the communication is most often at the junction between the proximal two-thirds and distal third of the leg [11]. Mahakkanukrauh and Chomsung found 5.9% in the popliteal fossa, 1.9% in the middle third of the leg, 66.7% in the lower third of the leg, and 25.5% at or just below the ankle [16]. In Albay et al.'s study the level union was at the distal third of the leg in 43% of the cases, at the middle third of the leg in 46% of the cases, and at the upper third of the leg in 11% of the cases [13]. In our study the majority of the cases (56.7%) showed union at the distal part of the leg, 36.7% in the middle 1/3 of the leg, and 6.6% in the popliteal fossa.  Table 4 summarizes the places of union between PCN and MSCN.

Length of the medial sural cutaneous nerve in Ortiguela et al.'s study is 21–33 cms, peroneal communicating nerve 20–38 cms, lateral sural cutaneous nerve 5–13 cms, and sural nerve 11–20 cms [6]. Mahakkanukrauh and Chomsung found medial sural cutaneous nerve length to be 17–31 cms and lateral sural cutaneous nerve 15–32 cms [16]. In our study medial sural cutaneous nerve length ranged from 6 to 43 cms, peroneal communicating nerve from 2.5 to 39 cms, lateral sural cutaneous nerve from 8.5 to 40 cms, and sural nerve from 3.5 to 39.5 cms.

## 5. Conclusion

The communication between peroneal communicating nerve and medial sural cutaneous nerve was seen in 60% of the specimens. The place of communication was often seen in the lower 1/3 of the leg. In most of the cases the peroneal communicating nerve was of larger calibre than that of the medial sural cutaneous nerve. Therefore in a situation requiring limited length of nerve graft material, the peroneal communicating nerve alone can be harvested and medial sural cutaneous nerve can be spared. The concept that sural nerve is purely sensory is changing. Many worked on this and showed that the nerve does contain motor fibres. It is recommended to screen the nerve electrophysiologically for motor fibres before nerve biopsy for interpretation of pathologic findings. Clinically the sural nerve is widely used for biopsy, nerve conduction studies, and nerve graft. The knowledge about the variation in the origin and course of sural nerve complex is important in evaluating sensory axonal loss in distal axonal neuropathies. All these variations should be borne in mind by clinicians and surgeons.

## Figures and Tables

**Table 1:** Formation of sural nerve.

Author	Number of cases	Typical sural formation	MSCN as SN	LSCN as SN
Bardeeen (1907) [8]	76	59.2%	39.4%	1.3%
William (1954) [3]	257	83.7%	15.9%	0.4%
Huelke (1958) [5]	198	80.3%	19.2%	0.5%
Ortiguela et al. (1987) [6]	20	80%	20%	—
Coert and Dellon (1994) [15]	50	96%	4%	—
Uluutku et al. (2000) [7]	40	77.5%	12.5%	—
Mestdagh et al. (2001) [11]	37	67.6%	24.3%	8.1%
Mahakkanukrauh and Chomsung (2002) [16]	76	67.1%	24.3%	—
Ugrenovic et al. (2005) [12]	100	58.5%	32.2%	—
Aktan Ikiz et al. (2005) [14]	30	60%	16.7%	—
Sekiya et al. (2006) [10]	31	74.2%	16.1%	—
Pyun and Kwon (2008) [17]	26	76.9%	15.4%	—
Albay et al. (2012) [13]	100	71%	20%	—
Our study	100	60%	39%	1%

MSCN: medial sural cutaneous nerve; LSCN: lateral sural cutaneous nerve; SN: sural nerve.

**Table 2:** Presence or absence of the medial and lateral sural cutaneous nerve.

Author	Number of cases	MSCN present	MSCN absent	LSCN present	LSCN absent
Bardeeen (1907) [8]	76	100%	—	100%	—
William (1954) [3]	257	99.4%	0.4%	—	—
Huelke (1958) [5]	198	100%	—	78%	22%
Ortiguela et al. (1987) [6]	20	100%	—	95%	5%
Coert and Dellon (1994) [15]	50	100%	—	96%	4%
Uluutku et al. (2000) [7]	40	100%	—	100%	—
Mestdagh et al. (2001) [11]	37	97.2%	2.7%	70.2%	29.7%
Mahakkanukrauh and Chomsung (2002) [16]	76	100%	—	—	—
Ugrenovic et al. (2005) [12]	100	100%	—	—	—
Aktan Ikiz et al. (2005) [14]	30	93.4%	6.7%	83.3%	16.7%
Sekiya et al. (2006) [20]	31	100%	—	—	—
Pyun and Kwon (2008) [17]	26	100%	—	—	—
Our study	100	100%	—	80%	20%

MSCN: medial sural cutaneous nerve; LSCN: lateral sural cutaneous nerve.

**Table 3:** The origin of peroneal communicating nerve.

Author	Number of cases	From the CPN	From or with the LSCN	Absent
Bardeeen (1907) [8]	76	9.2%	50%	40.8%
William (1954) [3]	257	93%	2.7%	4.3%
Huelke (1958) [5]	198	33.3%	47%	19.7%
Ortiguela et al. (1987) [6]	20	5%	95%	—
Coert and Dellon (1994) [15]	50	12%	84%	4%
Uluutku et al. (2000) [7]	40	72.5%	10%	12.5%
Albay et al. (2012) [13]	100	68%	3%	—
Our study	100	20%	40%	40%

CPN: common peroneal nerve; LSCN: lateral sural cutaneous nerve.

**Table 4:** Place of union between MSCN and PCN.

Author	Popliteal fossa	Upper 1/3 of leg	Middle 1/3 of leg	Distal 1/3 of leg
William (1954) [3]	Majority of the cases	—	—	—
Huelke (1958) [5]	—	—	—	75%
Ortiguela et al. (1987) [6]	—	—	—	Majority of the cases
Coert and Dellon (1994) [15]	12%	—	—	84%
Uluutku et al. (2000) [7]	—	—	81.8%	—
Mestdagh et al. (2001) [11]	—	—	Majority of the cases	
Mahakkanukrauh and Chomsung (2002) [16]	5.9%	1.9%	66.7%	25.5%
Ugrenovic et al. (2005) [12]	—	—	—	—
Aktan Ikiz et al. (2005) [14]	—	60%		10%
Pyun and Kwon (2008) [17]	—	—	34.6%	42.3%
Eid and Hegazy (2011) [2]	—	—	—	62%
Albay et al. (2012) [13]	—	11%	46%	43%
Present study	2%	—	20%	38%

MSCN: medial sural cutaneous nerve; PCN: peroneal communicating nerve.

## References

[1] Bannister L. H., Berry M. M., Collins P. (1995). *Gray’s Anatomy. The Anatomical Basis of Medicine and Surgery*.

[2] Eid E. M., Hegazy A. M. S. (2011). Anatomical variations of the human sural nerve and its role in clinical and surgical procedures. *Clinical Anatomy*.

[3] William D. D. (1954). A study of the human fibular communicating nerve. *The Anatomical Record*.

[4] Hollinshead H. W. (1958). *Anatomy for Surgeons, the Back and Limb*.

[5] Huelke D. F. (1958). The origin of the peroneal communicating nerve in adult man. *The Anatomical Record*.

[6] Ortiguela M. E., Wood M. B., Cahill D. R. (1987). Anatomy of the sural nerve complex. *Journal of Hand Surgery*.

[7] Uluutku H., Can M. A., Kurtoglu Z. (2000). Formation and location of the sural nerve in the newborn. *Surgical and Radiologic Anatomy*.

[8] Bardeeen C. R. (1907). Development and variation of the Nerves and musculature of the inferior extremity and of the neighboring regions of the trunk in Man. *American Journal of Anatomy*.

[9] Kim C.-H., Jung H.-Y., Kim M.-O., Lee C.-J. (2006). The relative contributions of the medial sural and peroneal communicating nerves to the sural nerve. *Yonsei Medical Journal*.

[10] Sekiya S.-I., Suzuki R., Miyawaki M., Chiba S., Kumaki K. (2006). Formation and distribution of the sural nerve based on nerve fascicle and nerve fiber analyses. *Anatomical Science International*.

[11] Mestdagh H., Drizenko A., Maynou C., Demondion X., Monier R. (2001). Origin and make up of the human sural nerve. *Surgical and Radiologic Anatomy*.

[12] Ugrenovic S., Vasovic L., Jovanovic I., Stefanovic N. (2005). Peculiarities of the sural nerve complex morphologic types in human fetuses. *Surgical and Radiologic Anatomy*.

[13] Albay S., Sakalli B., Kastamoni Y., Aydin Candan I., Kocabiyik N. (2012). Formation of the sural nerve in foetal cadavers. *Folia Morphologica*.

[14] Aktan Ikiz Z. A., Ucerler H., Bilge O. (2005). The anatomic features of the sural nerve with an emphasis on its clinical importance. *Foot and Ankle International*.

[15] Coert J. H., Dellon A. L. (1994). Clinical implications of the surgical anatomy of the sural nerve. *Plastic and Reconstructive Surgery*.

[16] Mahakkanukrauh P., Chomsung R. (2002). Anatomical variations of the sural nerve. *Clinical Anatomy*.

[17] Pyun S.-B., Kwon H.-K. (2008). The effect of anatomical variation of the Sural nerve on nerve conduction studies. *American Journal of Physical Medicine and Rehabilitation*.

[18] Amoiridis G., Schöls L., Ameridis N., Przuntek H. (1997). Motor fibers in the sural nerve of humans. *Neurology*.

[19] Ragno M., Santoro L. (1995). Motor fibers in human sural nerve. *Electromyography and Clinical Neurophysiology*.

[20] Sekiya S.-I., Tokita K., Banneheka S. K. (2006). Reexamination of the communicating branch between the sural and tibial nerves. *Journal of Anatomy*.

[21] Romanes G. J. (2008). *Cunningham’s Manual of Practical Anatomy*.

[22] Pimentel M. L., Fernandes R. M. P., Babinski M. A. (2005). Anomalous course of the medial sural cutaneous nerve and its clinical implications. *Brazilian Journal of Morphological Sciences*.

[23] George B. M., Nayak S. (2007). Sural nerve entrapment in gastrocnemius muscle—a case report. *Neuroanatomy*.

[24] Bryan B. M., Lutz G. E., O'Brien S. J. (1999). Sural nerve entrapment after injury to the gastrocnemius: a case report. *Archives of Physical Medicine and Rehabilitation*.

[25] Hwang K., Kim Y. J., Chung I. H., Won H. S., Tanaka S., Lee S. I. (2003). Innervation of calf muscles in relation to calf reduction. *Annals of Plastic Surgery*.

[26] Shaffrey M. E., Jane J. A., Persing J. A., Shaffrey C. I., Phillips L. H., Tindall S. C. (1992). Surgeon's foot: a report of sural nerve palsy. *Neurosurgery*.

[27] Bergman R. A., Thompson S. N., Afifi A. K., Saadeh F. A. (1988). *Compendium of Human Anatomical Variation*.

